# Scaling robotic surgery: the role, responsibilities and challenges of robotic proctorship in colorectal surgery

**DOI:** 10.1007/s11701-025-02444-9

**Published:** 2025-06-13

**Authors:** D. P. Harji, H. Mohan, R. Coates, D. Miskovic, C. Evans, R. J. Davies, J. Torkington, J. Khan

**Affiliations:** 1 Department of Colorectal Surgery, Manchester University NHS Foundation Trust, Manchester, UK; 2 Clinical Trials Research Unit, Leeds Institute of Clinical Trials Research, University of Leeds, Leeds, UK; 3 Department of Surgery, Peter MacCallum Cancer Centre, Melbourne, VIC Australia; 4 University of Melbourne, Melbourne, VIC Australia; 5 Sunderland Royal Hospital , Kayll Rd, Sunderland, SR4 7TP UK; 6 Imperial College, London, UK; 7 Department of Colorectal Surgery, St Mark’s Hospital, London, UK; 8 Department of Colorectal Surgery, University Hospital Coventry and Warwickshire, Coventry, UK; 9 Cambridge Colorectal Unit, Addenbrookes’ Hospital, Cambridge University Hospitals NHS Foundation Trust, Cambridge, UK; 10 University of Cambridge, Cambridge, UK; 11 Department of Colorectal Surgery, University Hospital of Wales, Cardiff, UK; 12 Department of Colorectal Surgery, Queen Alexandra Hospital, Portsmouth Hospitals NHS Trust, Portsmouth, UK; 13 School of Health and Care Professions, University of Portsmouth, Portsmouth, UK

**Keywords:** Robotics, Proctoring, Training

## Abstract

The adoption of robotic-assisted surgery (RAS) in colorectal procedures is growing rapidly, driven by advancements in technology and recognition of its clinical benefits. The dissemination of RAS technology relies heavily on robotic proctors, who are tasked with training and guiding their peers in adopting these advanced techniques. Despite their critical role, there is limited understanding of the training, responsibilities, and challenges faced by robotic proctors. A qualitative study was conducted using focus groups to understand the experiences, challenges, and training approaches of robotic colorectal surgery proctors in the UK and Ireland. Established proctors were invited to participate in the focus groups and to share insights into their practices, training methods, and the current state of robotic proctorship. The data were analysed thematically using NVivo software. Ten expert robotic surgeons participated in our study, with the majority working with Intuitive Surgical^®^ platforms (*n* = 9, 90%). Over 60% had been robotic trainers for more than 6 years and 60% were considered high-volume proctors, having proctored over 30 surgeons, and conducted more than 20 proctored cases annually. Thematic analysis revealed eight interconnected themes: proctor selection, proctor responsibilities, proctored training, accreditation, challenges, industry-proctor partnerships, emerging technologies, and network and support. Robotic proctoring is essential for scaling RAS adoption, however, it is a complex arena, with significant gaps in training frameworks and support systems. Establishing standardised guidelines and professional support structures is critical to ensure consistency, quality, and safety in robotic surgical training.

## Background

Robotic assisted surgery (RAS) continues to grow rapidly, with an annual reported growth of 15% globally and a total of 1.24 million cases performed across all surgical specialties in 2020 [1]. Within the field of colorectal surgery, the adoption of RAS continues to grow at pace across a range of robotic platforms, with a diversification of the indications beyond cancer resections [2]. The growing recognition of the clinical and patient benefits of robotic colorectal surgery, has led to industry partners to work collaboratively with thought leaders and clinical experts to develop and expand robotic colorectal surgery programmes.

Continuing professional development for established surgeons wishing to pursue RAS colorectal surgery is complex, due to the lack of broadly available and structured educational resources, limited widespread access to robotic platforms and simulators, competing professional and clinical priorities, and challenges associated with prolonged absences from surgical practice to participate in comprehensive training placements such as fellowships [3, 4]. To circumvent these challenges industry partners have designed multimodal robotic training programmes, consisting of theoretical online training, case observation, simulation, and proctored training [5]. Robotic training is delivered by established surgeons, selected by industry partners and are largely focussed on platform specific training and are remunerated for their time by industry partners. These established robotic surgeons assume the role of surgical trainers and are interchangeably referred to as proctors, preceptors, or coaches, depending on the individual robotic platform and industry partner.

Robotic training has evolved significantly over the last few years, with several professional organisations focussing their efforts on delivering standardised proctored training programmes for consultant surgeons [6, 7], with the development of procedure-specific curricula [8] and online training webinars. To date, the focus has largely been on robotic training programmes and their effectiveness, in terms of scaling up robotic colorectal surgery, with little focus on the workforce i.e. robotic proctors, delivering this training. Proctoring requires significant skill, expertise, and time. To upscale robotic surgery across the United Kingdom requires an in-depth understanding of the delivery of current proctored training to established surgeons including the characteristics of the workforce delivering this training, their training, responsibilities, and challenges.

## Methods

An in-depth qualitative study was undertaken using focus group methodology in November 2022. A convenience sampling strategy was employed to identify current colorectal surgery robotic proctors registered in the UK and Ireland with industry partners (Intuitive Surgical^®^ and Cambridge Medical Robotics^®^). All industry registered robotic colorectal proctors were invited to participate and requested to complete a short survey outlining individual robotic and proctoring experience.

An online focus group was held and facilitated by two dedicated facilitators (DH and HM). The aims of the focus group were reiterated at the start of the session and verbal consent was obtained from all participants. Ethical approval was waivered for this study. A structured topic guide informed the content of the focus group (Appendix II). This study has been reported in keeping with the standards for reporting qualitative research [SRQR] [9].

## Data analysis

The focus group recording was transcribed verbatim and was anonymised prior to data analysis. All transcripts were imported into NVivo 12 for data management and analysis. A framework method was employed for qualitative analysis [10]. The transcripts were coded line by line by one researcher, synthesising recurring ideas and concepts into codes. The coded outcomes that are sufficiently similar were grouped into similar categories and then themes. A detailed codebook was created during the transcription, this provided detailed definitions regarding codes to enable others to be able to easily interpret, and apply them to the raw data if necessary. All codes and themes were then discussed within the research team to ensure they formed a coherent pattern and to check whether the identified themes reflect the meanings evident in the dataset as a whole.

## Results

A total of ten expert robotic surgeons, working across the Intuitive Surgical^®^ (*n* = 9) and Cambridge Medical Robotics^®^(*n* = 1) platforms participated in the focus group. The majority of participating surgeons were expert surgeons having been practicing consultant surgeons for a significant period of time, with over 60% practicing as a robotic trainer for over 6 years, with significant annual volumes of individual robotic cases (Table 1). Half of the proctors had undertaken a specific robotic train the trainers’ course, with a third of proctors having a formal educational qualification (i.e. diploma or certificate of medical education). Most proctors were considered to be high volume proctors having proctored over 30 surgeons (*n* = 6, 60%) and undertaking more than 20 proctored cases per annum (*n* = 6, 60%).

**Table 1:** Proctor characteristics

Variables	Number (%)
No of years practicing as a consultant surgeon	
1	–
2–5	1 (10%)
6–10	4 (40%)
11–15	2 (20%)
16–20	2 (20%)
> 21	1 (10%)
No of years practicing as a robotic trainer	
1	1 (10%)
2–5	3 (30%)
6–10	4 (40%)
> 10	2 (20%)
No of robotic cases as console surgeon per annum	
< 10	–
11–25	1 (10%)
26–50	4 (40%)
51–75	4 (40%)
76–100	1 (10%)
Train the trainers course	
None	3 (30%)
Generic train the trainers	2 (20%)
Robotic train the trainers	5 (50%)
Laparoscopic train the trainers	–
Formal educational qualification	
Yes	3 (30%)
No	7 (70%)
Number of surgeons proctored	
< 10	3 (30%)
11–20	0 (0%)
21–30	1 (10%)
> 30	6 (60%)
Number of proctored robotic cases per annum	
< 10	2 (20%)
10–20	2 (20%)
20–30	4 (40%)
> 30	2 (20%)
Use of teleproctoring/remote proctoring	
Yes	4 (40%)
No	6 (60%)
Types of operations proctored	
Right hemicolectomy	7 (70%)
Right hemicolectomy (complete mesocolic excision)	4 (40%)
Anterior resection/abdominoperineal resection	7 (70%)
Subtotal colectomy	5 (50%)
Ventral mesh rectopexy	4 (40%)
Pelvic lymphadenectomy	1 (10%)
Other	4 (40%)
Proctoring/operative style	
1. Operate with the primary surgeon with joint console operating to illustrate learning points	2 (20%)
2. Advisory role	3 (30%)
3. Advisory role with console operating to address any intraoperative issues/challenges	5 (50%)
Patient contact	
Pre-operative introduction	2 (20%)
Consent with specific focus on the role of proctoring	0 (0%)
Intra-operative contact	8 (80%)

### Themes

**Fig. 1:**
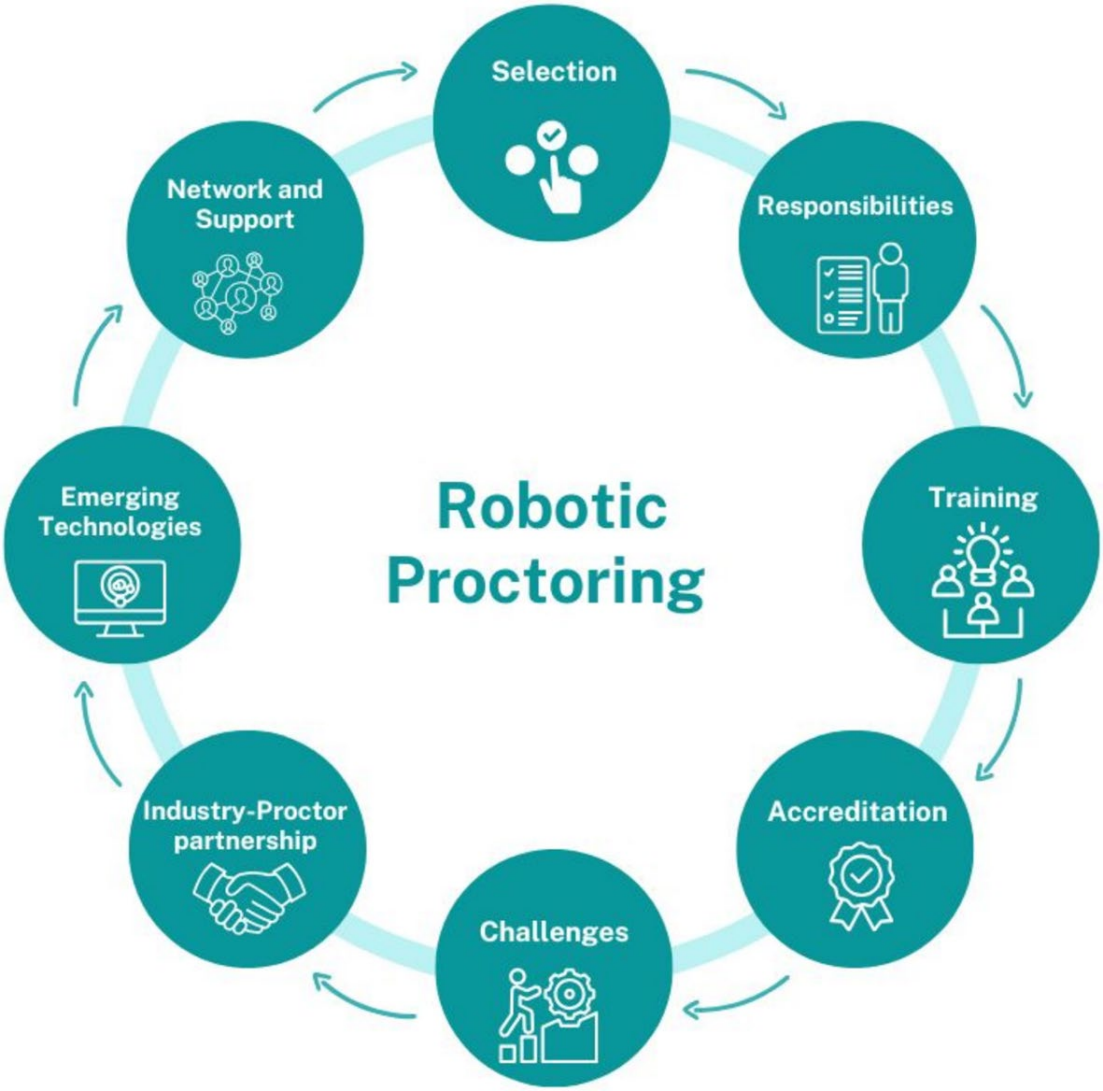
Themes relevant to robotic proctoring

A total of 180 unique codes were categorised into eight themes. These themes are proctor selection, proctor responsibilities, proctored training, accreditation, challenges, industry–proctor partnerships, emerging technologies, and network and support. These themes are interlinked and reflect the complexity of robotic proctoring within the UK (Fig. 1).

### Proctor selection

Proctor selection is currently undertaken and driven by industry partners and representatives, based on arbitrary criteria. The key considerations at the time of selecting proctors include number of robotic cases performed, robotic case-mix, overall robotic platform and clinical experience and co-existing industry relationships. Robotic surgeons who are over the learning curve and within the proficiency phase are generally selected to be proctors.

*‘…you have to be over the learning curve. The learning curve is around somewhere between 40 to 80 cases or 100 seems to be a safe figure... you have to have seen it all. You have to be comfortable with different techniques and then you can become a proctor’.*

There are no set selection criteria for proctors due to the evolving and dynamic clinical landscape of robotic surgery, with expanding clinical and operative indications coupled with new and emerging platforms coming to market. The increase in acquisition of new robotic systems and the associated need to train new surgeons, coupled with the limited pool of available robotic proctors, leads to broadening the selection criteria for proctors to fulfil the overall demand.

*‘...there is a huge uptake in robotics. The number of proctors is limited’*

### Proctor responsibilities

The industry appointed proctors assume the role of ‘robotic trainer’ to established consultant surgeons, with the main emphasis being of platform-related training. The overall aim is of peer-to-peer knowledge transfer regarding the use of the robotic platform, its utility, its key advantages, and appropriate tips and tricks. The aim of robotic proctoring is not to provide clinical or operative training. Therefore, the overarching responsibility of the proctor, from an industry perspective, is to ensure safe use of the robotic technology.

However, the proctors perceive their responsibilities to be broader than just platform related training, and assume they are three-fold; clinical, patient safety and training. The proctors pride themselves on delivering high quality operative training to enhance overall clinical care and prioritise patient safety. During proctored cases there is often an exchange of operative approaches, strategies and ideas between proctors and the training surgeon. The role of the proctor is not to dictate intraoperative strategy, guide operative decision-making or to perform component parts of the operation. However, there are occasions, whereby the training surgeons’ operative knowledge and/or ability do not allow them to complete a complex operative task on the robotic platform i.e. specific anastomotic technique. In these relatively rare instances, proctors do not feel it is appropriate for them to intervene operatively, as this is beyond their remit. However, in the interests of patient safety, they may encourage the training surgeon to assume their default operative approach i.e. laparoscopic or open surgery.

*‘If you feel that the person who's being trained on the system is putting the patient at risk by persevering with using that system because they cannot complete the operation (robotically) or perform a robotic technique, then they should convert to the technique that they know whether or not that's open or laparoscopic. And then your responsibility is to ensure patient safety…its taken me a while to come to terms with where my role in these things but I think for self-preservation, you have to be quite clear about it, your job is not to perform the operation or tell them to how to do something they are not competent to perform’.*

### Proctored training

Robotic proctoring is unique in that it involves delivering platform related training to established surgeons, who have been accredited through a national training programme and have an independent clinical practice. Training is often delivered in a variety of forms from case observations, dry and wet labs and proctored training on live cases. There is no formal competency based ‘curriculum’ or assessment underpinning proctored training. Training styles vary from proctor to proctor based on their individual experience and expertise and relationship with the training surgeon. Some proctors prefer to take a ‘hands-on’ approach, performing key components of the operation to demonstrate robotic technique, whereas others prefer to teach platform related skills alone. Some proctors meet the training surgeon prior to proctoring to establish shared objectives and develop an understanding of the training surgeons’ needs. Occasionally, the proctors maintained a relationship with training surgeons following the completion of proctoring to provide advice and guidance over a longer timeframe. Training styles and approaches were often personalised to the capabilities, skills and needs of the training surgeon.

*‘I find it very difficult when you are there to just have a verbal communication… sometimes you have to set it up for them to allow them to progress...and that's the job that you show them some tips and tricks and the moves that they can do to get from A to B, so keeping hands off is very difficult for me.’*

*‘We should accept that the people we are teaching are competent laparoscopic surgeons who are moving to a robotic platform. And so, my view is if that we are teaching the platform not the operation.’*

There are no specific training or educational requirements to become a robotic proctor, with no requirement for a formal qualification in training. Proctors tend to demonstrate interest in education and training, they have usually held posts as clinical or educational supervisors, and have significant enthusiasm for robotic training and education. Proctors are not required to complete feedback forms, have periodic mandatory performance reviews or go through a ‘revalidation’ process. Although there is no formal process for feedback and assessment, the majority of proctors collect their own clinical and proctoring data to ensure quality assurance.

*‘Quality assurance comes from our outcome data and your feedback from your trainees, this is collected for all (proctored) cases’.*

The proctors often find their scope of training extends beyond formal industry-based training, as they often provide training in-house to consultant colleagues or as part of wider fellowship training programmes. Their position as an industry appointed proctor often allows them to have a wider sphere of influence within the surgical community in the delivery and direction of robotic training due to their overall experience.

### Accreditation

Proctors believe their role is to ensure the safe dissemination of robotic technology through high quality training. As part of this process, they are expected to ‘sign off’ and accredit surgeons as being competent. Accreditation is a complex process, which ensures surgeons are appropriately trained and recognised for reaching a particular standard. The difficulty with ‘sign off’ and accreditation with the proctoring process is the small number of supervised cases i.e. 1–5 and the types of cases supervised. This is further complicated when there are multiple proctors training a single surgeon, leading to a lack of continuity in training standards and assessment. Difficulties in the sign off process arise when the basic robotic platform related competencies have been achieved, however, the overall robotic operative standard is considered to be suboptimal. In these instances, the proctors occasionally find it difficult to sign off training surgeons as individual entities responsible for robotic training and would like broader professional sign off. Overall, there is a lack of a standardised framework for sign off and accreditation for robotic surgery.

*‘…we've been asked to write an email to say that we are signing off for them to do the procedure. But I find this a really difficult and hard task because often I have only seen a few cases, and they are all different procedures…’*

### Proctor challenges

Proctors face a unique set of challenges, which are environmental, interpersonal, clinical, ethical, and medicolegal. The environmental challenges are related to working within an unfamiliar clinical setting, with an unknown theatre set up and team, and limited knowledge and understanding of the training surgeons’ operative capabilities and prior experience. Poor case selection by the training surgeon often compounds these challenges. Interpersonal challenges are related to differences in opinion, personality clashes between proctors and training surgeons, and the delivery of feedback. The lack of an objective and standardised process to deliver open and honest feedback to training surgeons can be challenging. Clinical challenges include complex or inappropriate case selections, unexpected intraoperative complications, and proctors needing to maintain their own clinical practice and balancing this with proctoring. The ethical and medicolegal challenges relate to patient safety, avoiding clinical harm, and unclear boundaries regarding overall responsibility for the patient. At present there is no clear guidance with regards to this, which can lead to proctors feeling uncertainty regarding their medicolegal position when difficult situations arise, with no official organisational or regulatory body to seek guidance from.

*‘The challenges are all significant...you go to an environment which is alien. The case selection is poor…the trainees don’t have enough overall colorectal experience or volume….and then there are issues that you have there with some personalities.’*

### Industry–proctor partnerships

Overall, the proctors view their relationships with industry partners as positive, welcoming the opportunity to teach and train consultant colleagues, develop new training skills and in having a broader impact on the adoption of robotic technology beyond their own hospital. This partnership between proctors and industry partners is considered to be essential in disseminating high-quality, peer-to-peer robotic training and knowledge transfer to novice surgeons. The proctors are broadly transparent with their relationship with industry partners and are happy to disclose this as appropriate.

The robotic proctors are selected and remunerated by industry partners to deliver platform related training. Given the financial relationship between the proctor and the robotic platform provider, the priorities and objectives of both parties are expected to align. Often, the robotic industry partners have already established a relationship with the hospital, prior to the commencement of proctoring, having been involved in the procurement process, and occasionally, in the selection of surgeons. There is no predefined selection criteria regarding institutional or individual surgeon volume or expertise prior to commencing a robotic colorectal program, which is often guided by the host hospital and relevant clinical leads. The proctors have no involvement in this process. The role of proctors is to execute the final part of the initial implementation process, by training the pre-selected surgeons. This can occasionally lead to difficulties, when the pre-selected surgeons are considered by the proctor to not be suitable for robotic training or to not have achieved an acceptable surgical standard.

Occasionally, there is an ‘expectation’ for proctors to showcase and demonstrate all key components and instruments associated with the robotic platform and promote these irrespective of the cost or financial implications. This can sometimes lead to a misalignment in objectives between proctors and industry due to differing priorities. The proctors believe they should be able to transfer their knowledge and skills to the training surgeon based on their own experience and robotic expertise and should not be expected to ‘sell’ or ‘showcase’ instruments not routinely used in their own practice.

*‘you have to work out where your loyalty lies… there have been instances when I’ve been expected to open five or six different instruments…but industry expects you to use X-Y instruments so that you can promote them…’*

### Emerging technologies

Robotic surgery is an evolving landscape with new platforms and technologies coming to market. This leads to further complexities for proctors as they navigate delivering training across new platforms and in new ways. Remote telementoring has been employed in robotic surgery to help deliver platform based and operative training. This is associated with unique challenges, including geographical location, communication, technological challenges, and patient safety. Delivering of remote telementoring must be carefully considered with this modality of training reserved for training surgeons with prior experience with the robotic platform and not for index cases, or for complex cases, whereby additional surgical support is considered valuable.

*‘We had to think about how we were going to deal with the trainee including how you teach on the system, especially when dealing with telementoring part, especially for the difficult cases and training and how you were going to do this safely.’*

The introduction of new platforms into the clinical arena will pose unique proctoring challenges, due to the limited overall experience with the new platform, unique technologies, and differing system designs i.e. modular versus mainframe. Proctor selection for new platforms is likely to be based on limited experience with the new platform, early adoption of the new platform, or established robotic surgeon on a different platform. Early adopters of new robotic platforms should be developed as robotic proctors of the future by sharing their experience and facilitate the onward dissemination of new technology. Established robotic proctors and surgeons should demonstrate agility in learning the intricacies and mechanics of new systems to help develop the robotic landscape further. Understanding the platform-to-platform interactions by working across multiple robotic systems will provide unique and valuable insights to the robotic surgical community and requires early adopters and experienced proctors to work together in tandem.

*‘…he was an early adopter, and has practiced quite early on without having done hundreds and hundreds of cases... he knows how to operate and how to use the equipment…so I think he should be developed as a proctor for the new system…’*

*‘We should be willing to learn when a new platform comes up. You may be an expert on robotics and a particular system, but when the system changes completely, you may have to unlearn certain things that you've done and be humble enough to learn the new technique.’*

### Network and support

There is a small pool of established robotic colorectal proctors within the United Kingdom, consequently, an informal network has developed to provide support and advice. The proctors use this network to discuss challenging cases, difficult proctoring sessions or when specific medicolegal issues may have arisen. Established proctors tend to provide informal mentorship and support to new and upcoming robotic proctors. There are several industry run forums for proctors to exchange ideas, collaborate and learn new teaching/training techniques. Overall, the proctors feel relatively well supported by robotic industry partners. There is, however, a sense of feeling of a lack of support from organisational bodies, such as surgical societies and associations. This is mainly due to the lack of guidance and regulation regarding the medicolegal and ethical aspects of proctoring.

‘*I have spent many hours speaking to other proctors about proctoring, cases, outcomes etc.….it is very useful.’*

## Discussion

Robotic proctoring facilitates the widespread dissemination of robotic colorectal surgery through peer-to-peer training and knowledge transfer across the United Kingdom, allowing the development of new programmes, upskilling of established surgeons, and increasing robotic access for patients. Proctorship is a complex phenomenon with several intersecting themes identified including proctor selection, responsibilities, and training. These themes are balanced against the challenges specific to proctoring, which include clinical, interpersonal, ethical, environmental, and medicolegal. Consequently, there is a clear need to support proctors in delivering high quality robotic training within unfamiliar environments and organisations. All these themes must be appropriately considered against the rapidly evolving landscape of robotic surgery, with new and emerging platforms coming to market, along with, new modalities of delivering training i.e. telementoring/teleproctoring.

The proctors, who participated in our focus group were early adopters of robotic surgery, who crossed over into robotic proctoring once foundational proficiency had been established. Our proctors were established consultant surgeons who had been practising as high-volume robotic proctors for a significant period, with the majority in surgical practice for over 6 years (*n* = 9, 90%), with proctoring experience of over 30 surgeons (*n* = 6, 60%) and undertaking more than 20 proctored cases per annum (*n* = 6, 60%). Proctor selection is currently informal and largely driven by industry partners, relying on factors such as case volume, case mix, and clinical experience, with little emphasis on educational or training background. While this pragmatic approach addresses immediate training demands, the lack of standardised criteria raises concerns about consistency and quality. Furthermore, the burgeoning adoption of robotic platforms has led to dilution of these criteria to meet increasing demand. This trade-off highlights the tension between expanding the proctor pool, whilst maintaining high quality training standards. Previous training programmes in colorectal surgery, such as Lapco for laparoscopic colorectal surgery training or the UK TaTME (Transanal total mesorectal excision) programme, had predefined, established criteria for the selection of proctors [11–13]. These programmes were underpinned with a strong theoretical foundation, pre-defined proctor and trainee selection criteria, and standardised feedback mechanisms. It is the lack of these key educational components within existing UK robotic proctoring programmes, which contribute to some of the challenges experienced by proctors, such as inappropriate surgeon or patient selection, lack of objective feedback and potential issues around accreditation. These shortcomings in industry-based proctorship programmes have been recognised by professional organisational bodies, such as the European Society of Coloproctology, who have developed high-quality, evidence-based, theoretically driven robotic colorectal surgery training programmes [8, 14, 15].

Current proctoring programmes lack a strong theoretical and educational foundation, which is evident in the variety of training styles employed and the variable amount of ‘hands-on’ assistance provided by individual proctors. The lack of formal competency-based curricula and objective assessment metrics further contribute to this. Our proctors preferred to take an advisory role (*n* = 8, 80%), with a proportion intervening if there were any arising intraoperative difficulties (*n* = 5, 50%). The term ‘robotic proctor’ is a misnomer, as in practice a range of training approaches are employed, including proctoring, precepting, mentoring and coaching. These are all distinct concepts which can be used to help train surgeons when adopting new technologies and techniques safely. However, there is a significant difference in the roles and responsibilities of the individual trainer depending on the training style adopted. The role of a proctor is to ‘observe and evaluate’, with the overarching aim of independent assessment of knowledge and skill, with reporting of this to a credentialing committee [3, 16]. In contrast, the role of the preceptor is to facilitate the acquisition of new knowledge through peer-to-peer knowledge transfer, ensuring the appropriate transfer of skills from within a simulated setting to a real world setting and assisting with key aspects of the procedure [3]. Mentoring aims to provide advice, guidance and solutions, whilst coaching focuses on continual improvement of performance [17]. These differing strategies need to be recognised and incorporated into a standardised framework to enhance the theoretical foundation of robotic proctoring and to ensure the delivery of high-quality, individualised, competency-based training. Recognising the array of training styles currently employed in robotic proctoring has important clinical, ethical, and medicolegal implications, due to the varying levels of ‘hands on’ training and operating provided by individual proctors.

There are several key challenges involved with proctoring which are widely recognised this includes time away from clinical practice, medicolegal implications of working across multiple institutions, ethical implications, maintaining expert skills, and continuing personal professional development [11, 18, 19]. We highlight further challenges including clinical challenges regarding patient selection, maintaining patient safety, interpersonal challenges, accreditation and sign off and industry-proctor relationships and terms of employment. Some of these challenges can be easily addressed with clear institutional guidelines on the scope of practice for proctors’ including overall responsibilities, authority limits and degree of patient interaction with appropriate consent. This needs to be standardised across all proctored programmes to ensure consistency in approach and to appropriately set the expectations of all key stakeholders, thus limiting any clinical, interpersonal, or patient safety issues. From a medicolegal perspective, the implications of an ‘active’ surgical intervention by a proctor are not clearly defined [20–22]. To mitigate for any medicolegal, indemnity or liability issue, the proctors are advised to ensure they hold the appropriate license to practise in the UK, ensure there is an honorary contract in place with the host institution, ensure patients are appropriately informed and consented of the proctored nature of the operation, ensure there is a predefined scope of intervention, with appropriate documentation and debriefing at the completion of the cases. These measure safeguard proctors, surgeons, hospital organisations and most importantly patients [21].

Our work highlights the contribution robotic proctors make to the adoption of robotic surgery through high quality peer-to-peer training. However, our work is limited by the unidimensional perspective employed in this study, with representative views sought from experienced proctors, with the majority working for Intuitive Surgical®. The inclusion of proctors from other companies, industry partners, and surgeons who were trained through the proctoring process, may have provided a multidimensional and richer qualitative insight into robotic proctoring. The limited pool of proctors available within the UK at the time of the focus group may have led to selection and reporting bias within our study. As the number of proctors expands within the UK, alongside the introduction of new of robotic systems, it is important to ensure the views of all key stakeholders are captured and incorporated into the development of any future guidance or framework to help support and expand robotic proctoring within the UK.

Our work provides key insights into the complexities of delivering of robotic training to established surgeons through proctoring supported by industry partners. There is a clear need to provide robust guidance and quality assurance for the delivery of high-quality training by proctors to support the accelerated adoption of robotic surgery in the UK, whilst ensuring appropriate support for proctors and maintaining patient safety. A structured framework or guidance supporting robotic proctors can mitigate the potential for harm and clinical risk which may occur during proctoring [20]. This will ensure transparent and standardised criteria for proctoring, enhance the educational foundations of proctoring, ensure robust feedback and accreditation processes. At present there is limited organisational support from professional bodies to help proctors navigate the challenges associated with robotic proctoring, with most of the support being informal from other established proctors or from the employing industry partners. There is an urgent need to address this, especially, as we continue to see the expansion and development of robotic technology within the UK and further afield. The key stakeholder organisations involved in the oversight and training of surgery in the UK, including specialty associations, such as the Association of Coloproctology of Great Britain and Ireland (ACPGBI) have a crucial role in taking this work forward and developing key standards for robotic proctoring.

## Data Availability

No datasets were generated or analysed during the current study.

## Appendix 1: Proctorship survey

Basic demographical data.

1. Year of consultant appointment
2. Number of years practising as a robotic colorectal surgeon
3. Number of years practising as a robotic proctor
4. How many robotic cases do you perform as the console surgeon per year?
5. Have you attended a train-the-trainers course?
6. How many surgeons have you proctored?
7. How many robotic cases do you proctor per year?
8. Where do you proctor?

UK Europe Other

9. Do you use teleproctoring?
  - If yes, on how many occasions have you used this?
  - Do you use teleproctoring in combination with face-to-face proctoring?
10. Types of robotic colorectal operations you proctor

Right hemicolectomy CME Subtotal colectomy LAR/APER

Pelvic sidewall lymphadenectomy Pelvic exenteration Ventral mesh rectopexy

## Appendix II: Topic guide for focus groups

1. Welcome and introduction including Chatham House rules
2. Proctorship Focus Group Questions
  i. What are the key requirements to become a robotic proctor?
  ii. What are the key challenges associated with being a robotic proctor?
  iii. How should robotic proctors be assessed? What quality assurance processes should be implemented to do this?
  iv. How should robotic proctors maintain their and develop their skills?
  v. How should professional organisations (e.g., ACPGBI) support robotic proctors?
  vi. Medico legal implications, responsibility of care and dealing with difficult situations
  vii. Any other comments
